# An American Text-Book of the Diseases of Children

**Published:** 1899-12

**Authors:** 


**An American Text-Book of the Diseases of Children.** By AMERICAN TEACHERS. Edited by LOUIS STARR, M.D., assisted by THOMPSON S. WESTCOTT, M.D. Second Edition. Pp. xvi., 604, 605–1244. London: The Rebman Publishing Co. (Ltd.). Philadelphia: W. B. Saunders. 1898.

Four years ago we had the pleasure of reviewing this excellent text-book on the diseases of children, when the first edition made its appearance. We have now before us the second edition, revised and enlarged. The old problem, whether a book containing twelve hundred pages should be published in one volume or two, appears to have been exercising the minds of the publishers, for this new edition, unlike its predecessor, appears in the latter form. Personally we have hitherto been in favour of the single volume wherever it was possible, but the far easier handling of the two volumes has in this instance at any rate almost converted us.

We remember, we trust now with regret, the reception, vigorous if curt as far as language was concerned, which we were from time to time apt to give to so-called *new* editions of medical books, when, with a belief born of inexperience that the newest treatment must of necessity be the best, we rushed to expend our hard-earned guineas, alas! only to find that our "new edition" contained nothing in addition to the old one, except perchance a few improvements in punctuation, a slight enlargement in the index, a fresh preface and a *later date*. The handsome work before us, however, is a "new edition" in the real sense of the word. The articles on typhoid fever, rubella, chicken pox, tuberculous meningitis, hydrocephalus, and scurvy have been entirely rewritten, whilst new articles appear on modified milk, lithæmia, and orthopædics, and more or less revision has been given to the chapters on infant feeding, measles, diphtheria, and cretinism. We are sorry to see, however, that the section on exercise and massage has been omitted from the new volumes, especially as we seem just now to be realising more than ever the value of physical exercise and the great importance of exercising and developing *all* the muscles of the body, and learning more and more about the use of massage, that great agent in "depriving rest of its evils."

In a book like that under consideration classification always presents a certain amount of difficulty, especially where diseases medical and surgical are considered in the same work. In this matter there has been some improvement, though the classification in the present volumes is by no means perfect. Tuberculosis and malaria have been quite rightly removed from their old places, and included in the section on infectious diseases; but we are puzzled to understand by what process of reasoning tracheotomy and intubation of the larynx are called "diseases of the respiratory system." We had hitherto looked upon them as methods either of relief or cure.

After these few adverse remarks, we feel that we should not be doing justice to this thoroughly trustworthy text-book if we did not call attention to one section that did not appear in the first edition. We allude to the admirable contribution on orthopædics by Dr. James E. Moore. It is contained in twenty-eight pages of text, well up to date, which are illustrated by some exceptionally good figures, of which we specially commend those representing congenital torticollis, early hip-joint disease, rachitic spine, and a little patient supporting his weight in the position so characteristic in dorsal Pott's disease. A large number of full-page plates, many of which are beautifully coloured, and a full and good index, complete two volumes which would make a valuable as well as handsome addition to the library of any medical practitioner.

